# Marine Spirotetronates: Biosynthetic Edifices That Inspire Drug Discovery

**DOI:** 10.3390/md17040232

**Published:** 2019-04-19

**Authors:** Alexander A. Braddock, Emmanuel A. Theodorakis

**Affiliations:** Department of Chemistry & Biochemistry, University of California San Diego, La Jolla, CA 92093-0358, USA; aabraddo@ucsd.edu

**Keywords:** natural product, spirotetronate, antibiotic, anticancer, drug resistance, Diels-Alder, Diels-Alderase, biosynthesis, total synthesis, Bcl-2 inhibitor, pABA inhibitor, UPR apoptosis

## Abstract

Spirotetronates are actinomyces-derived polyketides that possess complex structures and exhibit potent and unexplored bioactivities. Due to their anticancer and antimicrobial properties, they have potential as drug hits and deserve further study. In particular, abyssomicin C and tetrocarcin A have shown significant promise against antibiotic-resistant *S. aureus* and tuberculosis, as well as for the treatment of various lymphomas and solid tumors. Improved synthetic routes to these compounds, particularly the class II spirotetronates, are needed to access sufficient quantities for structure optimization and clinical applications.

## 1. Introduction

It has been estimated that every milliliter of seawater contains about 1 million bacteria [1,2,3,4]. To thrive in such a competitive environment, microbes need to produce secondary metabolites, also referred to as natural products, which primarily serve to regulate bacterial communication or thwart predators. Endowed by a rapid growth rate and an uncanny ability to exchange genetic material, bacteria constantly evolve. Such a rapid evolution goes hand-in-hand with a highly adaptable biosynthetic machinery to ensure survival of the fittest [5]. As predators acquire resistance to certain secondary metabolites, the preyed upon synthesize more sophisticated compounds to ensure their defense [6,7]. Thus, from a chemistry perspective, the structures of natural products are dynamic and undergo continuous refinement in order to address the challenges faced by the producing bacteria [8,9,10]. 

In Western medicine, there are numerous examples of bacterial natural products that have been used as drugs or have become starting points for the development of new drugs [11]. Arguably the best example is penicillin. Soon after its discovery in 1928 [12], penicillin revolutionized the use of antibiotics in medicine and led to the development of several drug classes still in use today [13]. Unfortunately, the golden era of antibiotics, sometime between the 1940s and the 1970s, was rather short-lived as antibiotic resistance started to occur [14,15,16,17,18]. In fact, to-date antibiotic resistance is responsible for about 23,000 deaths annually in the United States alone, and another 25,000 deaths in Europe [10,11,19]. Alarmingly, as the number of antibiotic-resistant bacteria has proliferated, the number of new antibiotics and antibiotic-based research has dwindled. This troubling phenomenon is a combination of stringent FDA requirements, deprioritization by the pharmaceutical industry and over-prescription of antibiotics [20]. Similarly to bacterial resistance, cancer cells also become tolerant and refractory to previously efficacious pharmacological treatment. [21]. These precipitous developments of multidrug resistance necessitate the need for second-line anticancer drugs [22]. As with antibiotics, marine natural products have played an important role in the development of new anticancer strategies with some being adopted clinically [23]. In turn, this has led to an urgent need for the discovery of new antibiotics from underexplored sources such as marine bacteria [24,25,26,27,28]. 

In the past decade, natural products have been revisited as viable drug leads [18,19,29,30,31,32,33]. The renewed interest in natural products as drug leads is a combination of the improved ability for high-throughput screening of natural sources and the failure of combinatorial chemistry to deliver sufficient numbers of viable drug candidates [34,35]. Among natural products, those derived from marine sources have been valuable contributors to human health, with many being approved for clinical application [36,37]. This review seeks to bring attention to the large family of natural products known as spirotetronates, which have broad potential as pharmaceutical leads.

## 2. Spirotetronates: Derivation, Classifications, and Biological Relevance

**Figure 1:**
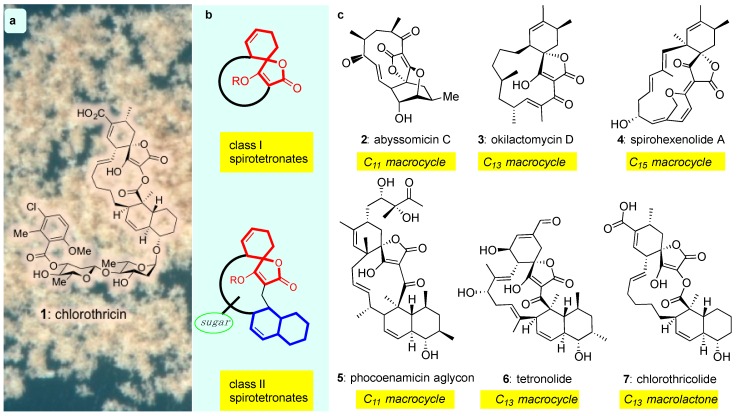
(**a**) Chemical structure of chlorothricin (**1**) the archetype of the spirotetronate natural products; (**b**) General motif of class I and class II spirotetronates; (**c**) Representative examples of class I (**2**–**4**) and class II (**5**–**7**) spirotetronates.

The discovery of chlorothricin (**1**) in 1969 [38] defined a new class of bacterial metabolites that possess an architecturally intriguing structure and promising chemotherapeutic properties as antitumor antibiotics. Referred to as spirotetronates, these compounds are commonly produced by both marine and terrestrial actinomyces and are structurally identified by the presence of a tetronic acid spiro-linked to a cyclohexene (or a cyclohexane) ring (Figure 1b, red motif). Spirotetronates can be further divided into two sub-classes: class I, which contain the spirotetronate motif integrated within a macrocycle and class II, which also contain an integrated decalin (Figure 1b, blue motif) and frequently, oligosaccharide chains attached to the decalin and/or the macrocycle (Figure 1b, green circle) [39,40]. Beyond these general requirements, the structures of class I and II are highly variable (Figure 1).

The spirotetronates are prepared by their source organisms for use as antibiotics in interbacterial warfare. Many have been found to display broad-spectrum activity against Gram-positive bacteria [41,42,43]. Some spirotetronates have also been described as effective chemotherapeutic agents and recently, retroviral reactivators [44,45]. The disparate biological impact of spirotetronates warrants their exploration as drug candidates. The remainder of this review will focus on the contextualized recent advances in the fields of class I and II spirotetronates, and how they support the need for improved access via synthesis. 

## 3. Class I Marine Spirotetronates

Among the marine class I spirotetronates, none have enjoyed more attention than the abyssomicins [46]. Originally derived from *Verrucosispora maris* AB 18-032, abyssomicin C (**2**) was isolated from a sediment sample from the Japanese Sea at a depth of 283 m along with two biologically inactive relatives (abyssomicins B and D) [47]. Named for the extreme depth at which its source resides, abyssomicin C and many other members of the family have been found to be potent inhibitors of the *para*-aminobenzoic acid (pABA) pathway via their action as chorismate mimics [47].

### 3.1. Biosynthesis

**Figure 2:**
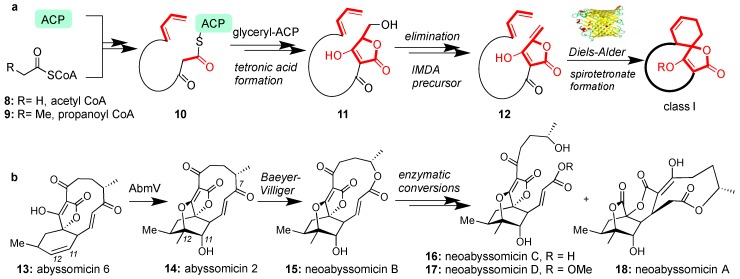
(**a**) Generic biosynthetic pathway for class I spirotetronates; (**b**) Enzymatic conversion and postulated biosynthetic relationship of various abyssomicins.

The biosynthesis of all class I spirotetronates begins through a series of iterative chain elongation steps in type I polyketide synthase (PKSI) by attaching acetyl CoA (**8**) and/or propanoyl CoA (**9**) to the acyl carrier protein (ACP) [48,49]. After reaching the appropriate length (e.g., structure **10**), the process is terminated by a glyceryl-ACP unit [50] to form, after condensation, tetronic acid (**11**). The tetronic acid motif then undergoes acetylation-elimination to form butenolide 12 [51]. Intramolecular Diels-Alder (IMDA) cyclization between the butenolide and the pendant diene yields the general architecture of the class I spirotetronates (Figure 2a). Recent studies have unveiled an enzyme responsible for catalyzing the IMDA reaction to produce abyssomicin C (**2**) [52]. The spirotetronate cyclase, AbyU is a homodimeric Diels-Alderase found to catalyze the construction of the spirotetronate motif of **2** in one of its two eight-stranded antiparallel β-barrels [52]. This active site is however not conserved in the biosynthesis of class II spirotetronates [52].

Conversion of abyssomicin 6 (**13**) to abyssomicin 2 (**14**) is biosynthetically accomplished by AbmV, a P450 enzyme responsible for C11-C12 tandem epoxidation and SN2-epoxide opening sequence (Figure 2b) [53]. Analogous enzymes are proposed to oxidize other abyssomicins but AbmV is the first isolated enzyme and is relevant to the newly discovered neoabyssomicins [54]. In this case, a Baeyer-Villiger oxidation at the C7 carbonyl group is postulated to expand the macrocycle and provide neoabyssomicin B (**15**) [54]. Neoabyssomicin B could then be enzymatically converted to neoabyssomicins C and D (through hydrolysis or methanolysis), and neoabyssomicin A (through a retro-aldol/Michael reaction sequence, Figure 2b) [54]. The enzymatic pathway to these natural products has yet to be elucidated, presenting opportunities to advance our understanding of the biosynthesis of this family [55].

### 3.2. Synthesis

**Figure 3:**
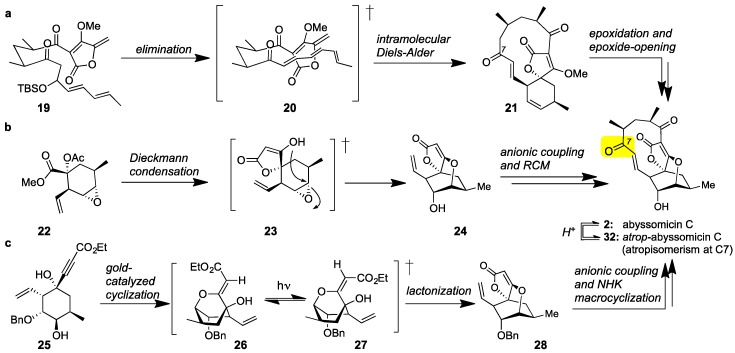
(**a**) The Sorensen bioinspired synthesis of abyssomicin C (**2**) featuring an IMDA reaction; (**b**) The Nicolaou atroposelective synthesis of abyssomicin C and *atrop*-abyssomicin C (**32**, Figure 5) featuring a ring-closing metathesis (RCM); (**c**) The Saicic synthesis of *atrop*-abyssomicin C featuring a Nozaki-Hiyama-Kishi (NHK) macrocyclization.

Class I marine spirotetronates and specifically abyssomicin C (**2**) have been the subject of extensive synthetic studies [56,57,58,59]. The first synthesis of **2** was executed by the Sorensen group [60]. This daring synthesis features a bioinspired Diels-Alder reaction to convert acyclic tetronate **19** to spirotetronate **21**. Epoxidation of the cyclohexene ring and epoxide-opening then formed abyssomicin C (**2**) together with its C7 atropisomer (later identified as atrop-abyssomicin C (**32**), Figure 3a). More recently, the Nicolaou group reported an atroposelective synthesis of abyssomicin C [61,62,63]. Central to this strategy is the construction of spirotetronate motif (**24**) via a Dieckmann condensation of **22** [64]. Chain elongation and Grubbs-catalyzed macrocyclization then yielded **2**. The observed atroposelectivity toward **2** was attributed to the stereoselectivity of the macrocyclization (Figure 3b). These authors also reported that interconversion of abyssomicin C (**2**) to its atropoisomer (**32**) can be accomplished under acidic conditions. The Saicic group also synthesized *atrop*-abyssomicin C targeting spirotetronate (**28**) as the key intermediate [65]. To this end, cyclohexane **25** was converted to **28** under [(PPh_3_)AuNTf_2_]-catalysis [66]. Following chain elongation, a Nozaki-Hiyama-Kishi (NHK) reaction was employed to affect macrocyclization (Figure 3c). This strategy has also been applied to the synthesis and biological evaluation of various desmethylated versions of abyssomicin C [67].

### 3.3. Biological Relevance

**Figure 4:**
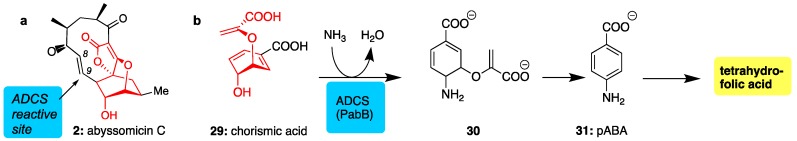
(**a**) Structure of abyssomicin C (in red is shown the conserved motif between chorismate and **2**); (**b**) *para*-aminobenzoic acid (*p*ABA) pathway showing amination of chorismate through the action of ADCS, the target of abyssomicin C.

Abyssomicin C (**2**) was found to be the first structure-based inhibitor of amino deoxychorismate synthase (ADCS), an enzyme essential for the conversion of chorismic acid (**29**) to p-aminobenzoic acid (pABA, **31**). Since the pABA pathway is exclusive to bacteria and essential to their survival, it represents an attractive target for antibacterial drug design [68,69]. Acting as a structural mimic of **29** (see common red motif, Figure 4), abyssomicin C binds at the chorismate active site of ADCS where it irreversibly reacts with a proximal cysteine via conjugate addition at the C9 center (Michael-acceptor) [70]. Unfortunately, this type of conjugate addition reaction is not very selective leading to off-target complications [71]. 

**Figure 5:**
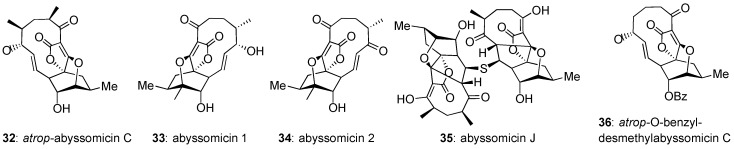
Relevant and recent members of the abyssomicin family.

In general, abyssomicins that do not contain the C8–C9 enone functionality are biologically inactive. An interesting exception is abyssomicin J (**35**, Figure 5), which is active (MIC = 3.125 µM) against *M. tuberculosis* [72]. It has been shown that 35 undergoes P450 oxidation in vitro, followed by a retro-Michael addition to yield abyssomicin C (**2**). In turn, this suggests a viable prodrug strategy for the masking of the Michael acceptor site of abyssomicin C to increase its biological half-life [72]. In addition, the Saicac group was able to increase the selectivity of atrop-abyssomicin C for Gram-positive bacteria over HeLa and PMBC (cancerous and healthy) cell lines through global demethylation and protection of the pendant alcohol as a benzyl ether [67]. Biological evaluation of this derivative, named atrop-*O*-benzyl-desmethylabyssomicin C (**36**), suggests that the methyl groups of native abyssomicin C play a role in the ligand-binding mode of its mammalian targets, whereas they are not critical for its antibiotic activity. Combining the properties of abyssomicin J (**35**) and atrop-*O*-benzyl-desmethylabyssomicin C (**36**) could lead to potent and selective antibiotics.

Abyssomicin 2 (**34**) was discovered as part of a natural products screening campaign to identify HIV-1 reactivators [45]. It was found that 34 reactivates HIV-1 at 56% at an EC_50_ of 13.9 µM. The mechanism of action of abyssomicin 2 is novel and remains to be elucidated. While conducting this study, the researchers also proposed a structural correction to abyssomicin 1 (**33**), showing it to be almost enantiomeric to the originally proposed structure, with the exception of the alcohol assignment [45]. The retroviral induction potential of abyssomicin 2 (**34**) demonstrates the family’s broad application as drug leads, not limited to antitumor antibiotics.

## 4. Class II Marine Spirotetronates

### 4.1. Biosynthesis

**Figure 6:**
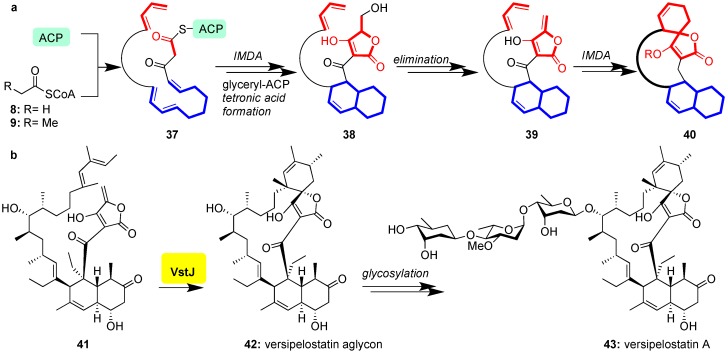
(**a**) General biosynthesis of class II spirotetronates; (**b**) VstJ-catalyzed intramolecular Diels-Alder reaction of **41** to **42** en route to versipelostatin A (**43**).

Class II spirotetronates are synthesized analogously to their class I counterparts (Figure 3). In this case, however, an intramolecular Diels-Alder (IMDA) produces the decalin moiety of **38** prior to chain termination of the PKSI and formation of the tetronate of **40** (Figure 6a) [73,74,75,76]. Recently VstJ, the Diels-Alderase responsible for spirotetronate-formation in versipelostatin (**43**), has been identified through comparison of genes responsible for versipelostatin synthesis with those of the biosynthetic cluster of chlorothricin, tetrocarcin A, and lobophorin, rationalizing conservation of the biosynthetic pathway [77]. When incubated with the Diels-Alder precursor **41**, VstJ induced stereoselective formation of **42** (Figure 6b); without VstJ this cyclization failed to occur [77]. Historically, this study provided the first example of conclusive enzymatic involvement in spirotetronate cyclization, although VstJ remains to be structurally characterized and its precise mechanism of catalysis is currently unknown. 

### 4.2. Synthesis

**Figure 7:**
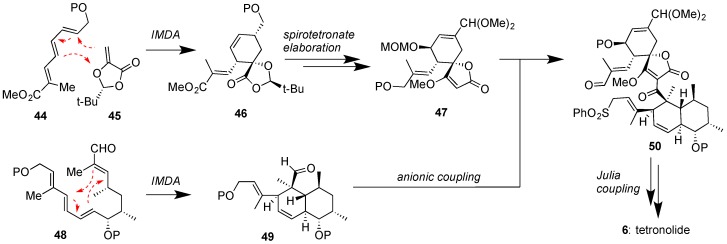
Roush and Yoshii groups’ approaches to tetronolide (**6**), featuring IMDA reactions for the construction of both the spirotetronate and the decalin motifs and a Julia coupling to ultimately close the macrocycle (P = various protecting groups).

To-date no total synthesis of a class II spirotetronate has been achieved. Since most class II family members contain one or two oligosaccharide chains, synthetic studies have targeted the synthesis of their aglycons [78]. On this front, the total syntheses of chlorothricolide (**7**) [79] and tetronolide (**6**) [80,81,82], the aglycons of chlorothricin (**1**) and tetrocarcins (51–54) respectively, have been reported. The key steps toward the synthesis of 6, as accomplished by the Yoshii group [80] and later improved upon by the Roush group [81], are shown in Figure 7. Both strategies feature a biomimetic IMDA reaction to convert **48** to **49**. The Roush approach to perform an IMDA reaction between **44** and **45** provided expedient access to spirotetronate motif 47. Coupling of 47 with 49 under Yoshii’s conditions afforded 50 that underwent macrolactonization under Julia coupling conditions to produce tetronolide (**6**). Similar strategies were subsequently reported for the synthesis of chlorothricolide (**7**) [79]. More recently, the Boeckman group reported an improved 27-step synthesis of tetronolide (**6**) using a similar IMDA and Julia-based key steps [82].

The IMDA reaction is prevalent in almost every synthetic study of class II spirotetronates [81,83,84]. All these efforts toward class II spirotetronate aglycons are too cumbersome to practically bring forward large quantities of material, and they neglect the oligosaccharide chains, which are essential for biological activity in all marine-derived class II spirotetronates [85]. No total syntheses of class II spirotetronates have been accomplished and the field would benefit from updated and more practical preparations of these natural products.

### 4.3. Biological Relevance

**Figure 8:**
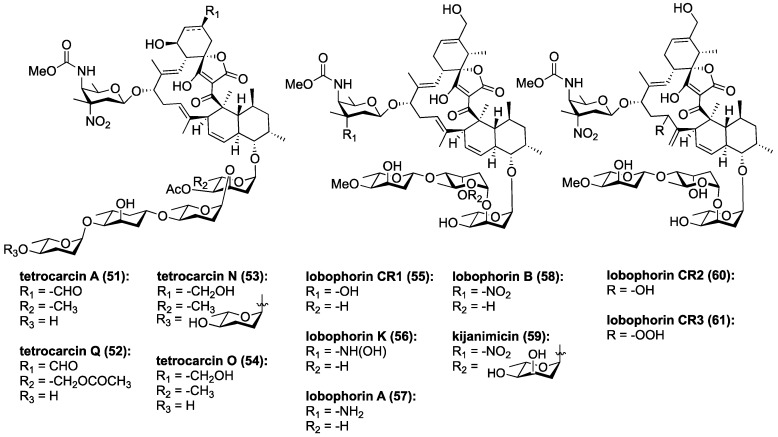
Chemical structures of selected tetrocarcins, lobophorins and kijanimicin.

**Figure 9:**
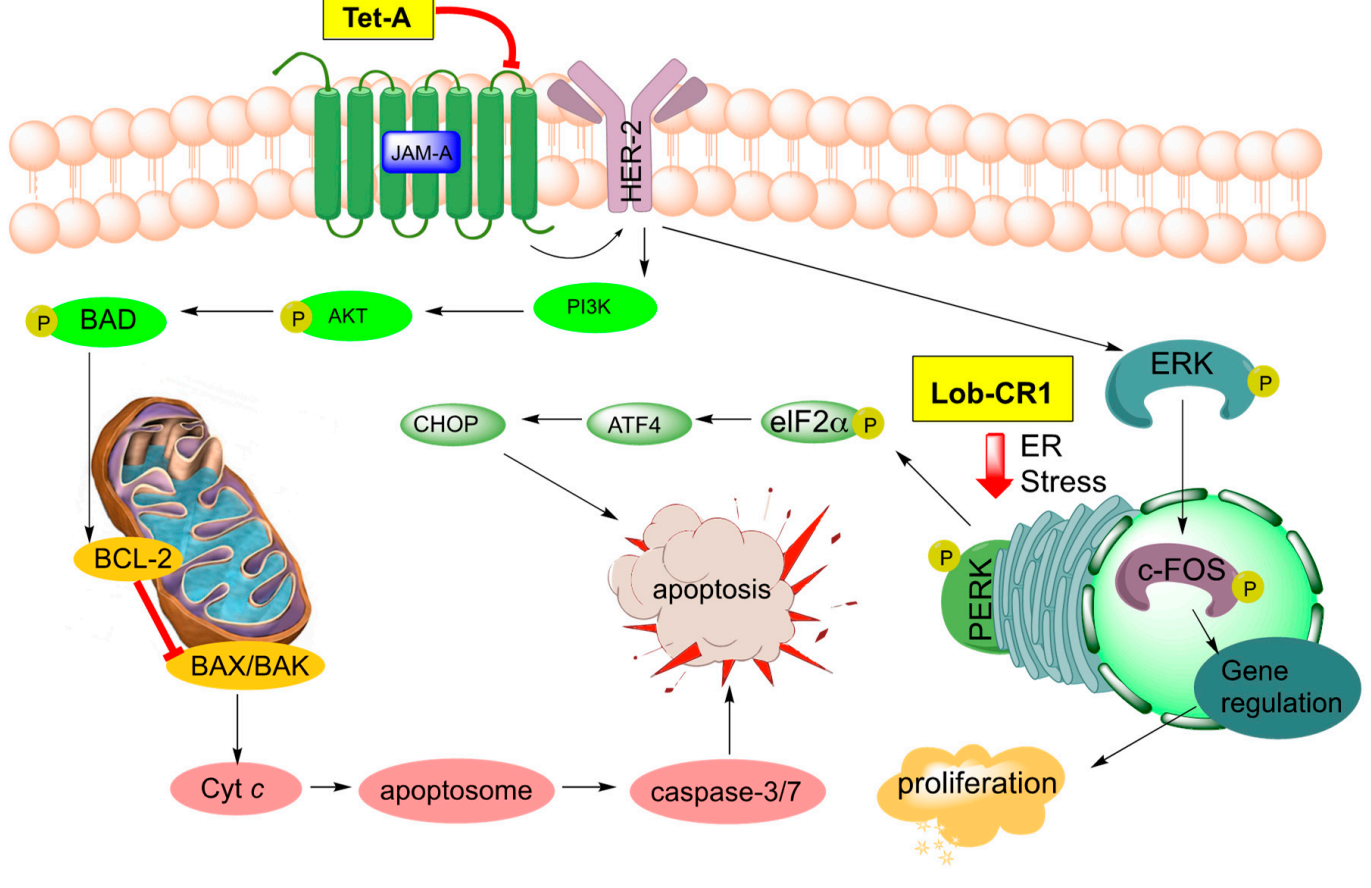
Signaling pathways targeted by tetrocarcin A (Tet-A, **51**) in breast cancer and recently discovered lobophorin CR1 (Lob CR1, 55) in oral carcinoma.

Tetrocarcin A (**51**, Figure 8) has been found to effectively inhibit Gram-positive bacteria by blocking RNA and protein synthesis [86]. In human cells, **51** was found to inhibit the integral intracellular membrane protein Bcl-2 and activate stress signals in the mitochondria and endoplasmic reticulum [44,87]. Responsible for the downregulation of caspase-3, which is an effector of mammalian cell death, Bcl-2 becomes overexpressed on the mitochondrial surface of cancer cells and is responsible for their anti-apoptotic properties [88,89,90,91,92]. Tetrocarcin A (**51**) was later found to directly induce apoptosis in breast cancer cells accompanied by activation of a proteolytic cascade of caspases and a concomitant decrease in phosphorylation of protein kinase B, pyruvate dehydrogenase kinase-1, as well as phosphatase and tensin homolog (Akt, PDK-1, and PTEN, respectively) [93]. Recently, the upstream target of tetrocarcin A (**51**) was found to be junctional adhesion molecule-A (JAM-A), a regulator of human epidermal growth factor receptor-2 (HER2) protein expression in breast cancer cells [94,95]. Downregulation of JAM-A was proposed to lead to inhibition of Bcl-2 and extracellular signal-regulated kinases (ERK), in turn resulting in suppression of proliferation and caspase-dependent apoptosis in breast cancer cells (Figure 9) [94]. This most recent study of tetrocarcin A (**51**) specifically advocates for its clinical application as a chemotherapy agent. 

Three new members of the lobophorin family have recently been used to trigger cell death in murine tumor fibroblasts through the apoptotic arm of unfolded protein response [96]. Lobophorins CR1 (**55**), CR2 (**60**), and CR3 (**61**) were isolated from *Streptomyces sp.* 7790_N4 from marine sediment in Costa Rica (Figure 8). Lobophorins CR1 (**55**) along with known lobophorins A (**57**), B (**58**), E and F [97] (not pictured) were found to induce endoplasmic reticulum (ER) stress leading to PERK-mediated phosphorylation of eukaryotic initiation factor 2 alpha (elF2α) in oral squamous cell carcinoma. In turn, this leads to activation of the proapoptotic transcription factors, ATF4 and CHOP, ultimately inducing CHOP-dependent apoptosis (Figure 9) [96]. These new members of the lobophorin family were identified using a screen targeting natural products that induce apoptosis through unfolded protein-response. Unfortunately, the limited cultivability of these lobophorins prevented the evaluation of lobophorins CR2 and CR3 [96]. Lobophorin B (**58**) is nearly identical to kijanimicin (**59**) [60,98], lacking an additional glycosylation at the B-sugar. Lobophorins CR2 and CR3 (**60**, **61**) appear to be allylic oxidation products of **58**, suggesting a biosynthetic relationship that merits further exploration. Additionally, lobophorin K (**56**) was isolated from *Streptomyces* sp. M-207 found in marine sediment at a depth of 1800 m, and was found to have cytotoxic activity against pancreatic carcinoma and breast adenocarcinoma, as well as moderate and selective antibiotic activity against *S. aureus* [99]. 

Recently three new marine tetrocarcins were discovered. Tetrocarcins N (**53**) and O (**54**) were isolated from *Micromonospora sp.* 5-297 using a PCR-based screen that targets glycosidic antibiotics [100]. They exhibited antibacterial activity against *B. subtilis*. Compound **53** was substantially more potent than **54** suggesting that extended oligosaccharide chains enhance the biological activity. Both **53** and **54** were less active than tetrocarcin A (**51**), suggesting that the formyl group at the spirotetronate motif is biologically important [100]. The most recent tetrocarcin to be discovered is tetrocarcin Q (**52**), which was isolated from *Micromonospora carbonacea* LS276 and contains an unusual acetoxy unit on its oligosaccharide motif [101]. 

**Figure 10:**
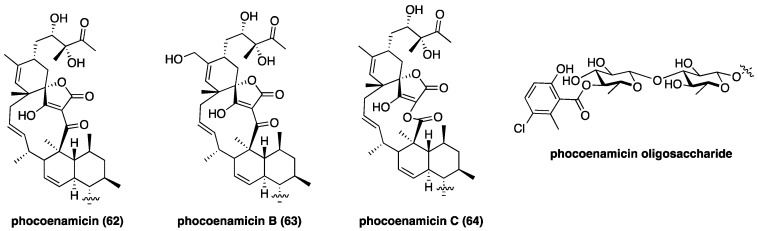
Recently discovered phocoenamicins with a novel C11 aglycon.

Phocoenamicin (**62**, Figure 10), a new class II aglycon with an uncommon C11 macrocycle, was discovered in the intestinal microbiome of a freshly deceased dolphin using a high-throughput antibacterial screen [102]. Compound **62** possesses potent activity against *Clostridium difficile* with a MIC of 2.6 µM compared to vancomycin’s 2.9 µM [102]. The structurally related phocoenamicins B (**63**) and C (**64**) were isolated from *Micromonospora* sp. and were found to be active against *S. aureus* [103]; phocoenamicin C (**64**) was also found to inhibit *M. tuberculosis*. In parallel to the chlorothricin family, **64** contains a lactone macrocycle that presumably derives from Baeyer-Villiger-type ring expansion of **62** [73]. The oligosaccharide motif of phocoenamicins remains unchanged throughout this family and features an unusual chlorosalicyclic ester. The new discovery of phocoenamicins reveals the untapped depth of the class II spirotetronate family, and their potential as drug leads.

## 5. Conclusions

In the ongoing public health crises of antibiotic resistance and untreatable cancers, marine natural products provide inspiration for the development of new drugs [104,105]. Among them, spirotetronates, products of bacterial warfare, deserve particular attention not only for their exquisite biosynthesis, but also for their exceptional promise as antitumor antibiotics. In particular the abyssomicins, representative members of the class I spirotetronate family, have been the subject of extensive studies related to their biosynthesis, chemical synthesis and mode-of-action. These studies have demonstrated the significant antibacterial potential of these compounds by targeting the pABA pathway. Nonetheless, challenges related to their indiscriminate reactivity and target selectivity need to be overcome before clinical evaluation. Class II spirotetronates are less studied, but many members have encouraging antibiotic and antitumor activity and interesting but unexplored mode-of-action. For instance, tetrocarcin A has been found to interfere with the Bcl2 pathway inducing apoptosis in cancer cells. The therapeutic relevance of this pathway has been recently demonstrated with Venetoclax, to-date the only approved drug that targets Bcl2 [106,107,108,109,110]. On the other hand, oligosaccharide chains attached to a class II aglycon are essential to antibiotic activities. Unfortunately, the overall complexity of the class II spirotetronates and the recalcitrance of their producers toward large-scale culturing, severely limits the availability of these compounds for further preclinical and clinical studies. In turn, this makes particularly significant and timely the development of improved synthetic [111,112,113,114,115,116,117,118,119,120] and biosynthetic strategies [121,122,123,124,125,126] to access class II spirotetronates. Research along these lines as well as the continuous exploration of marine bacteria [127,128,129,130,131,132] will define new paradigms for drug discovery.
